# Obstructive Sleep Apnea-Hypopnea Syndrome (OSAHS) in Patients With Acromegaly in Colombia

**DOI:** 10.7759/cureus.77557

**Published:** 2025-01-16

**Authors:** Alin Abreu Lomba, Juan Manuel Montoya Ospina, David Alexander Vernaza Trujillo, Santiago Sierra Castillo, David Aristizabal Colorado, Gildardo Mauricio López Osorio, Luis Fernando Guerrero Gonzalez, Doly Pantoja Guerrero, Henry M Arenas Quintero, Alejandro Alberto Castellanos Pinedo, Alex Valenzuela Rincón, Alejandro Pinzón Tovar

**Affiliations:** 1 Endocrinology, Imbanaco Clinic, Cali, COL; 2 Interinstitutional Group of Internal Medicine 1 (GIMI1), Universidad Libre, Cali, COL; 3 Epidemiology, Fundación Universitaria del Área Andina, Bogotá, COL; 4 Epidemiology, CES University, Medellín, Medellín, COL; 5 Interinstitutional Group on Internal Medicine 1 (GIMI1), Universidad Libre, Cali, COL; 6 Pulmonology, Universidad Libre, Cali, COL; 7 Pulmonology Department, Universidad del Valle, Cali, COL; 8 Endocrinology, Departmental University Hospital of Nariño Pasto, Pasto, COL; 9 Endocrinology, Clínica comfamiliar Pereira, Pereira, COL; 10 Endocrinology, Hospital Escuela Jose de San Martín, Buenos Aires, ARG; 11 Endocrinology, Universidad del Rosario, Bogotá D.C, COL; 12 Endocrinology, Universidad Surcolombiana, Neiva, COL

**Keywords:** acromegaly complications, cardiovascular risk (cvr), gh, heart disease, igf -1, osahs, polysomnography

## Abstract

Background

Acromegaly is a rare, chronic, and progressive disorder characterized by excessive secretion of growth hormone (GH) after the closure of the epiphyseal plates. The disease has an estimated annual incidence of 5 cases per 1,100,000 individuals. Sleep apnea/hypopnea syndrome (OSAHS) affects up to 80% of individuals with acromegaly and is recognized as an independent risk factor for the development of cardiovascular disease. This study aims to estimate the prevalence of SAHS and its associated factors in patients with acromegaly in Colombia.

Methods

This observational, retrospective cohort study utilized the National Registry of Patients with Acromegaly (RAPACO) data. The study evaluated patients who had undergone baseline polysomnography as a criterion for inclusion.

Results

A total of 163 patients were included in the study, of whom 89 (54.6%) were diagnosed with OSAHS. Women accounted for 99 (60.7%) of the cohort, aged 34.4 to 63.54 years. Patients with OSAHS exhibited a higher BMI and a longer disease duration. Additionally, they demonstrated elevated levels of IGF-1 (936.2 ± 447.2) and baseline GH (20.0 ± 20.4), both of which were statistically significant (p = 0.006 and p = 0.027, respectively). Severe apnea was the most prevalent form of the condition, and microadenoma was the predominant tumor type. Multivariate analysis identified disease duration and IGF-1 levels as the primary variables associated with OSAHS.

Conclusion

OSAHS is a prevalent comorbidity in patients with acromegaly, with hormonal factors playing a critical role in its pathogenesis. Consistent with previous studies, our findings demonstrate that elevated IGF-1 and GH levels are associated with greater OSAHS severity. Routine polysomnography (PSG) is recommended following the diagnosis of acromegaly. If OSAHS is confirmed, appropriate treatment should be initiated, and follow-up PSG should be performed during acromegaly management.

## Introduction

Acromegaly is a rare, chronic, and progressive disorder characterized by excessive secretion of growth hormone (GH) after the closure of the epiphyseal plates. A GH-producing pituitary adenoma primarily causes it and is commonly associated with elevated insulin-like growth factor I (IGF-I) levels. The most frequent clinical manifestations include acral and soft tissue overgrowth, joint pain, diabetes mellitus, hypertension, and both cardiac and respiratory failure [1,2].

The hypersecretion of GH and IGF-I contributes to craniofacial abnormalities and soft tissue overgrowth in the upper respiratory tract, predisposing patients with acromegaly to significant respiratory disturbances, present in up to 25% of cases [3]. These respiratory impairments result from various anatomical changes, including airway alterations, rib cage abnormalities, weakened muscular structures, and reduced chest and lung elasticity. Furthermore, upper airway obstruction is exacerbated by macroglossia, prognathism, thickened lips, and hypertrophy of the laryngeal mucosa and cartilage. These changes lead to symptoms such as excessive snoring, hypoventilation, and hypoxemia, often due to central and/or obstructive apneas [4].

These structural and functional changes significantly increase the prevalence of obstructive sleep apnea/hypopnea syndrome (OSAHS), a condition characterized by repeated episodes of airflow reduction or cessation during sleep [5]. These alterations lead to anatomical changes that promote airway collapse. Consequently, apneas and hypopneas cause repeated episodes of oxyhemoglobin desaturation, resulting in intermittent hypoxia, brief awakenings that fragment sleep, and significant fluctuations in intrathoracic pressure due to increased inspiratory effort [5]. The gold standard for diagnosing OSAHS is polysomnography, which records nasal and oral airflow, inspiratory effort, arterial oxygen saturation, snoring, pulse, and body position. This test detects apnea and hypopnea episodes and establishes a diagnosis when the apnea-hypopnea index (AHI) exceeds five events per hour [6].

OSAHS is associated with systemic inflammation, endothelial dysfunction, and dysglycemia, contributing to an increased risk of cardiovascular and respiratory diseases [7]. Globally, the prevalence of OSAHS ranges from 4% to 30%, depending on factors such as region, sex, age, diagnostic criteria, and severity measures [8]. This variability is also reflected in acromegaly patients, where OSAHS prevalence has been reported to range from 20% to 80%, as noted by Pivonello et al. [9]. Similarly, Vouzouneraki et al. observed prevalence rates between 44% and 87.5% in patients with active disease and 35% to 58% in those with controlled disease [10]. Other studies have reported similar OSAHS prevalence in patients with active and inactive acromegaly; however, significant reductions in AHI have been documented after treatment [11].

In Colombia, the estimated prevalence of acromegaly suggests that 1,440 to 2,880 cases could be diagnosed based on a prevalence rate of 30 to 60 cases per million individuals. However, official incidence rates remain undefined [12]. The Colombian Registry of Patients with Acromegaly (RAPACO), initiated in 2010, currently includes 201 patients, 60% of whom are women, with a mean age of 49.5 years. The average weight and BMI of registered patients were 75.1 ± 12.98 kg and 28.11 ± 4.33, respectively. The most common comorbidity was arterial hypertension (HTN), affecting 50.3% of patients, with a higher prevalence in women. Hypopituitarism was significantly more frequent in men. Disorders of carbohydrate metabolism were reported in 26.38% of patients, and cardiomyopathy was identified in 31.6% via echocardiography. Additionally, OSAHS was confirmed in 55.2% of patients evaluated with baseline polysomnography, with severe OSAHS present in 36.7% of these cases [13].

Although somatostatin analogs have been shown to effectively reduce GH and IGF-I levels [14], there is currently no evidence to suggest that these biochemical improvements correlate with better polysomnography parameters [15]. Furthermore, no studies have specifically evaluated the impact of these treatments on OSAHS improvement in Colombian patients with acromegaly.

Given that OSAHS is associated with a 10-year increase in cardiovascular risk in patients with acromegaly [16], this study aims to evaluate the behavior of OSAHS in this population. Specifically, it seeks to identify determinants of OSAHS in patients with acromegaly and explore the potential association between OSAHS and heart disease.

## Materials and methods

This observational, multicenter, retrospective single-cohort study included 163 patients selected from the national RAPACO registry. Eligible participants were patients aged 16 years or older with a confirmed diagnosis of acromegaly, baseline type 1 polysomnography recordings, and measurements of IGF-1 and GH levels. Type 1 polysomnography is considered the gold standard for diagnosing sleep disorders, including obstructive sleep apnea-hypopnea syndrome (OSAHS). It involves overnight monitoring in a sleep laboratory and includes measurements of brain activity, eye movements, muscle activity, heart rhythm, and breathing patterns. The diagnosis of OSAHS was defined as an apnea-hypopnea index (AHI) greater than five events per hour. Apnea was defined as the cessation of airflow for more than 10 seconds. Hypopnea was defined as a reduction in airflow between 30% and 90% for more than 10 seconds. The severity of OSAHS was classified as mild (AHI 5-15), moderate (AHI 15-30), or severe (AHI >30).

Patients with incomplete medical records were excluded from the study. Data collection was conducted across nine participating centers nationwide, with the local endocrinologist at each center responsible for gathering information from all relevant cases. All cases from these centers meeting the inclusion criteria were included in the analysis to ensure comprehensive coverage and minimize selection bias.

Data quality was assessed through an exploratory analysis of the dataset in Excel, where study variables were thoroughly reviewed. Statistical analyses were performed using IBM SPSS software version 29.0. Quantitative variables were evaluated for homoscedasticity using Levene's test, followed by the Kolmogorov-Smirnov test to assess data normality. Based on the results, group comparisons were performed using the Student’s t-test for normally distributed data and the Mann-Whitney U test for non-normally distributed data. Further group comparisons were conducted using analysis of variance (ANOVA) for normal distributions and the Kruskal-Wallis test for non-normal distributions.

For categorical variables, Pearson's Chi-square test was employed to compare proportions between groups. Logistic regression analyses were performed to explore potential associations, including subgroup analyses to control for confounding factors. Statistical significance was set at a p-value < 0.05, and 95% confidence intervals were calculated for all analyses.

## Results

**Figure 1:**
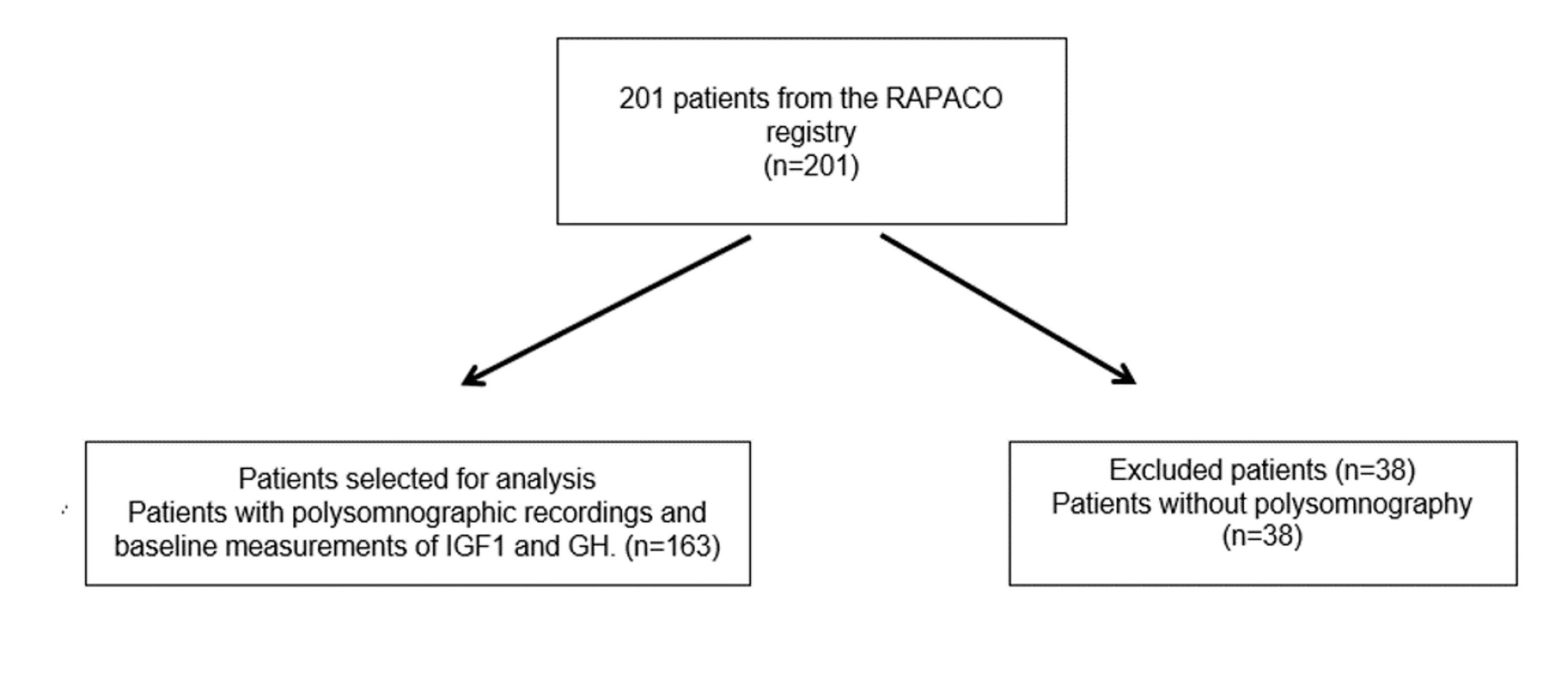
Selection Diagram of the Population Studied

This study included 163 patients who underwent type 1 polysomnography (Figure 1), of whom 89 (54.6%) were diagnosed with OSAHS. Female patients comprised 99 (60.7%) of the cohort, outnumbering males. The mean age of the patients was 50.0 years (range: 38.0-58.2), including both sexes. Patients diagnosed with OSAHS were frequently classified as overweight based on their body mass index (BMI) (Table 1). The average disease duration was 6.9 ± 4.2 years.

**Table 1:** Demographic and Clinical Characteristics of the Study Population M: mean; SD: standard deviation; Me: median; P25: 25th percentile; P75: 75th percentile; IGF-1: insulin-like growth factor 1; GH: growth hormone; ng/mL: nanograms per milliliter.

Variable	Mean (Standard Deviation), Median (P25–P75)
Age (years) (p25 - p75)	50.0 (38.0 – 58.2)
Evolution time (years) (M, SD)	6.9 (4.2)
BMI (kg/m²) (M, SD)	28.2 (4.5)
Cervical Perimeter (cm) (M, SD)	36.5 (4.8)
IGF-1 (ng/mL) (p25 - p75)	854.2 (582.0 – 1102.0)
Basal GH (ng/mL) (M, SD)	18.6 (23.6)

Regarding the severity of OSAHS, the severe category was the most prevalent, affecting 30 patients (20%), followed by moderate and mild cases, which were less common. Among the associated pathologies, macroadenoma was the predominant tumor type, observed in 130 (79.8%) of cases, compared to microadenoma (Table 2).

**Table 2:** Relationship Between Sociodemographic and Anthropometric Variables in Patients With Acromegaly PSG: Polysomnography. Cardiomyopathy: left ventricular hypertrophy (LVH); biventricular hypertrophy (BVH); valvulopathy; left ventricular hypertrophy with valvulopathy; not reported.

Variable	n (%)
Male	64 (39.3)
Female	99 (60.7)
Patients with PSG	163 (100.0)
Snoring	5 (3.1)
Mild apnea	26 (16.0)
Moderate Apnea	30 (18.4)
Severe Apnea	33 (20.2)
Macroadenoma	130 (79.8)
Microadenoma	33 (20.2)
Cardiomyopathy	52 (31.9)

Patients diagnosed with OSAHS had a longer average disease duration compared to those without OSAHS, with a mean of seven years (range: 1.0-27.0); however, no statistically significant differences were observed between the two groups (p > 0.05). Conversely, patients diagnosed with OSAHS were significantly older on average (p = 0.017) (Table 3).

**Table 3:** Relationship Between Demographic and Clinical Variables in Patients With OSAHS and Acromegaly M: mean; SD: standard deviation; Me: median; P25: 25th percentile; P75: 75th percentile; IGF-1: insulin-like growth factor 1; GH: growth hormone; ng/mL: nanograms per milliliter.

Variable	With OSAHS (n = 89)	Without OSAHS (n = 74)	p-value (f-value)
Evolution time (years) (P25–P75)	7.0 (1.0–27.0)	5.0 (1.0–20.0)	0.113
Age (years) (M, SD)	51.4 ± 13.6	45.9 ± 13.6	0.017 (0.893)
IGF-1 (ng/mL) (M, SD)	936.2 ± 447.2	756.6 ± 301.2	0.006 (10.310)
Basal GH (ng/mL) (p25 - p75)	14.0 (0.0–150.0)	10.3 (0.5–205.0)	0.027
Cervical Perimeter (cm) (M, SD)	37.4 ± 4.3	35.4 ± 5.1	0.306

**Figure 2:**
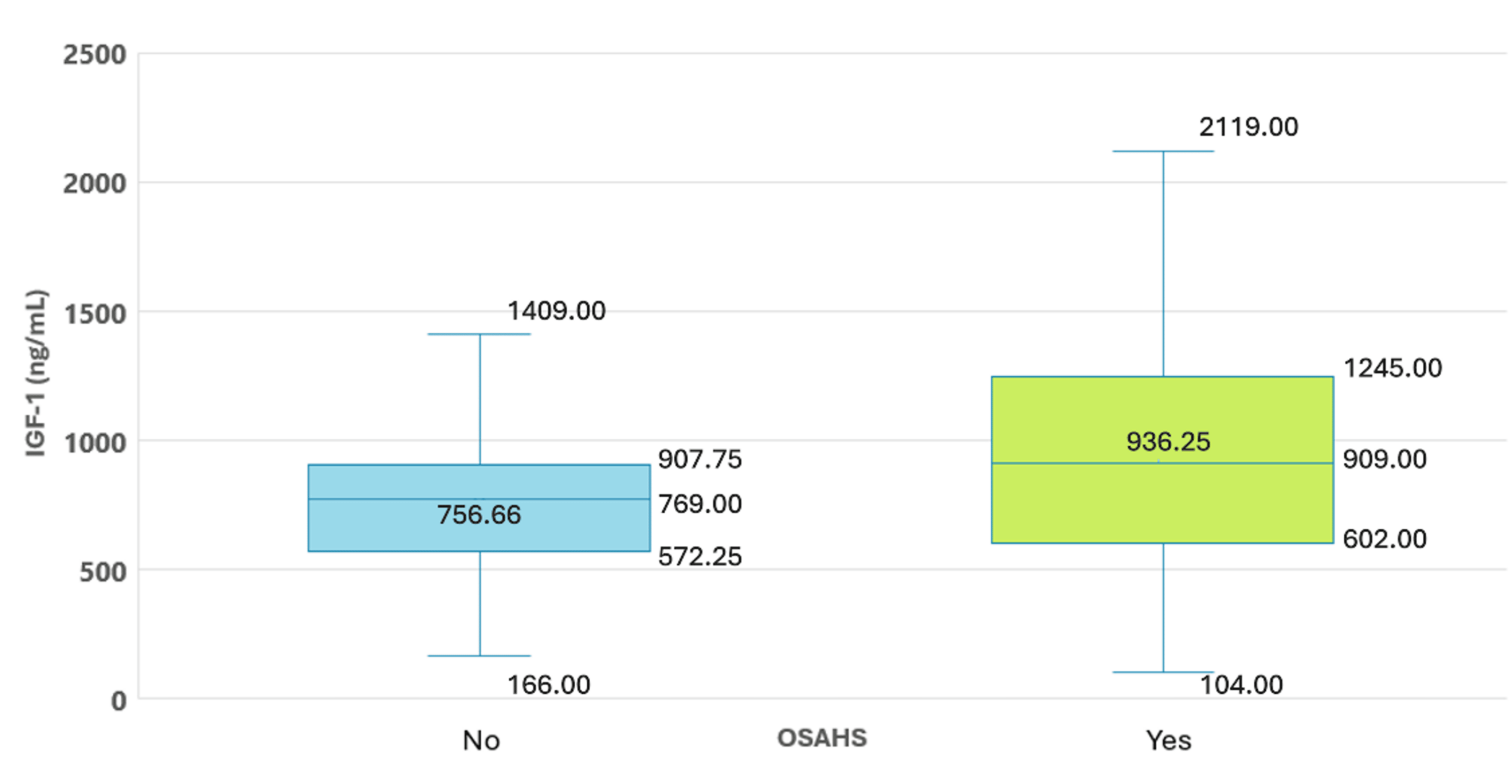
IGF-1 Levels in Patients With Acromegaly With and Without OSAHS IGF-1: insulin-like growth factor 1; OSAHS: obstructive sleep apnea/hypopnea syndrome.

**Figure 3:**
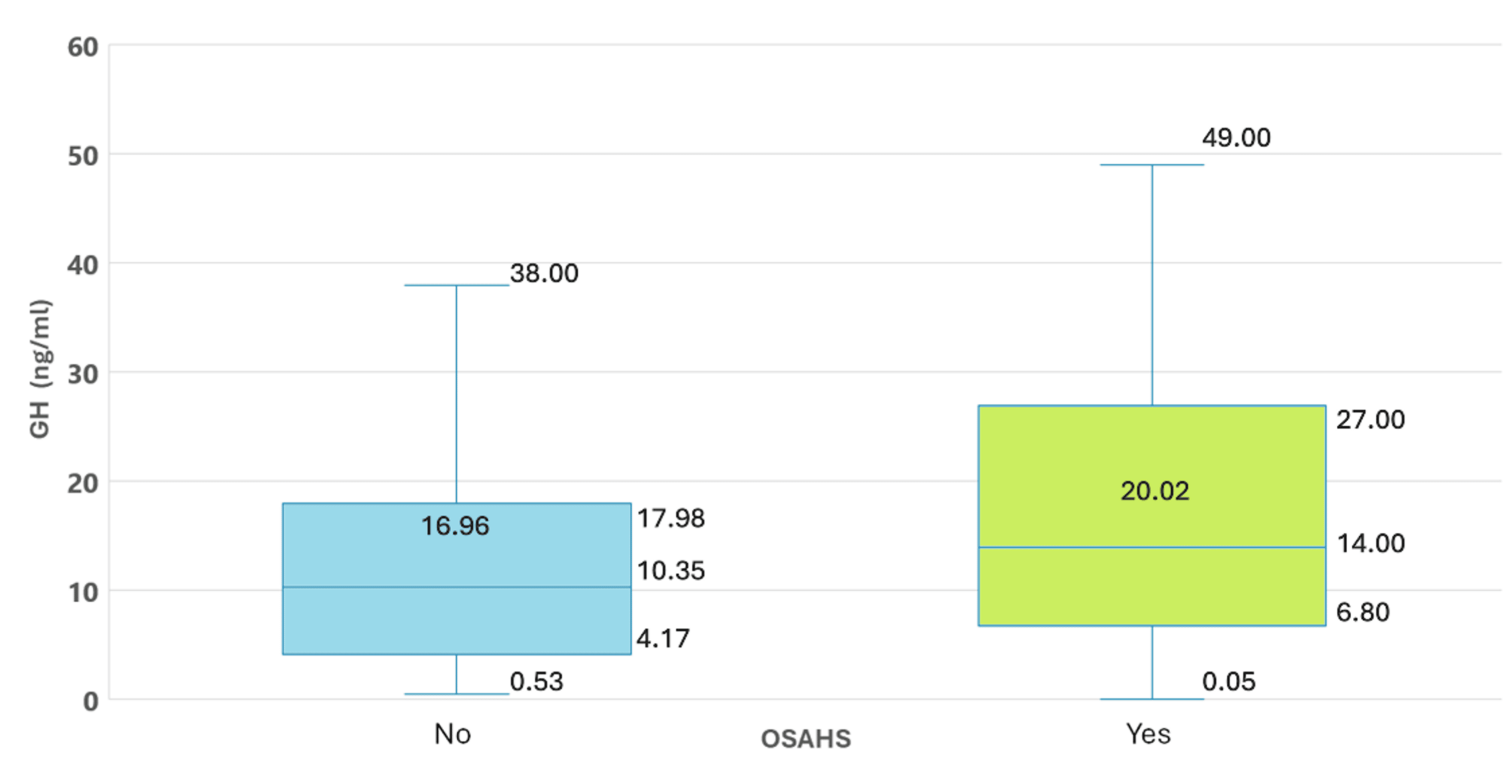
GH Levels in Patients With Acromegaly With and Without OSAHS GH: growth hormone (ng/mL); OSAHS: obstructive sleep apnea/hypopnea syndrome.

Patients with OSAHS also exhibited higher IGF-1 levels (ng/mL) compared to those without OSAHS, with an average of 936.2 ± 447.2 ng/mL, showing a statistically significant difference (p = 0.006) between the groups (Figure 2). Similar findings were noted for baseline GH levels, which were significantly higher in patients with OSAHS (p = 0.027) (Figure 3).

Regarding cervical circumference, patients with OSAHS had a significantly larger average circumference of 37.4 ± 4.3 cm, further demonstrating statistical significance.

In terms of OSAHS severity, patients with mild OSAHS had a shorter disease evolution time compared to those with moderate and severe OSAHS, with the severe group showing the longest median duration. A clear relationship was observed between OSAHS severity and IGF-1 levels, as higher severity corresponded to increased levels. Patients with severe OSAHS exhibited the highest IGF-1 levels, averaging 1034.3 ± 518.9 ng/mL, surpassing those in the moderate and mild categories. Similarly, baseline GH levels were elevated in patients with moderate and severe OSAHS, with the highest levels recorded in the moderate group.

Cervical circumference also showed a positive association with OSAHS severity, as patients in more severe categories presented larger diameters. A statistically significant difference in cervical circumference was found between the mild and moderate groups. No statistically significant differences were observed for other variables across the severity groups (Table 4).

**Table 4:** Relationship Between Clinical Variables and Severity of OSAHS in Patients With Acromegaly M: Mean; SD: Standard deviation; *Median (P25–P75); IGF-1: insulin-like growth factor 1; GH: growth hormone; ng/mL: nanograms per milliliter.

Variable	Mild Apnea (M ± SD)	Moderate Apnea (M ± SD)	Severe Apnea (M ± SD)	p-value
Evolution time (years)	6.6 (3.6)	7.5 (1–27.0)*	8.0 (1–25.0)*	0.377
Age (years)	51.8 (15.8)	51.7 (14.8)	50.8 (15.2)	0.964
IGF-1 (ng/mL)	793.8 (342.5)	951.7 (423.5)	1034.3 (518.9)	0.092
Basal GH (ng/mL)	11.0 (2.9–44.0)*	18.9 (11.9)	14.0 (0.0–150.0)*	0.572
Cervical Perimeter (cm)	35.0 (30.0–46.0)*	37.7 (4.1)	38.9 (4.1)	0.002

In the multivariate model aimed at identifying the variables that best explained the association between acromegaly and OSAHS, confounding factors such as age and BMI did not demonstrate a significant relationship with the presence of OSAHS. However, disease evolution time was significantly associated with OSAHS (p = 0.017), with an odds ratio (OR) indicating a 0.15-fold increase in the likelihood of apnea. IGF-1 levels (ng/mL) also showed a significant association with OSAHS (p = 0.047), although the odds ratio (OR = 1.001) did not indicate an increased risk (Table 5).

**Table 5:** Multivariate Model of Factors Associated with OSAHS in Patients with Acromegaly IGF-1: insulin-like growth factor 1; GH: growth hormone; BMI: body mass index; CI: confidence interval; cm: centimeters; ng/mL: nanograms per milliliter.

Variable	OR	95% CI	p-value
Age (years)	1.020	0.993 – 1.049	0.148
Evolution time (years)	1.155	1.026 – 1.300	0.017
Cervical circumference (cm)	1.109	0.990 – 1.242	0.073
IGF-1 (ng/mL)	1.001	1.000 – 1.002	0.047
Basal GH (ng/mL)	0.999	0.982 – 1.016	0.884
BMI	1.022	0.926 – 1.129	0.665

## Discussion

Acromegaly is characterized by excessive growth hormone (GH) secretion, leading to changes in soft tissues and bones and triggering systemic comorbidities such as diabetes mellitus, hypertension, heart failure, and OSAHS [11]. In this study, a prevalence of OSAHS of 54.6% was observed in patients with acromegaly who underwent baseline polysomnography during follow-up in the RAPACO registry. OSAHS was classified as mild in 16%, moderate in 18.4%, and severe in 20.2% of patients. The occurrence of OSAHS in acromegaly is thought to result from upper respiratory tract soft tissue hypertrophy, including the mucosa and macroglossia [17].

The prevalence observed in our study aligns with the 62.9% prevalence reported by Turan et al. in the Turkish population [18], but it is lower than the 74.1% found by Wolters et al. in the Dutch population, where patients were beginning medical treatment followed by surgery [19]. Our study population had an average BMI of 28.2 ± 4.5, which is consistent with the BMI averages of 29.7 ± 3.8 and 30.28 ± 5.41 reported by Turan and Wolters, respectively [18,19].

Regarding hormonal values, our study found an average IGF-1 level of 936.2 ± 447.2 ng/mL and a baseline GH level of 14.0 (0.0-150.0) ng/mL. Comparatively, Turan et al. reported lower IGF-1 and GH levels of 810.79 ± 262.36 ng/mL and 9.4 ± 2.4-32.6 ng/mL, respectively [18]. Additionally, our study showed a higher prevalence of acromegaly in women, consistent with data from the Colombian consensus on acromegaly [20]. Interestingly, unlike the general population, where OSAHS is more common in men [21], in our study, 60.7% of patients with OSAHS were women, and only 39.3% were men.

Age and disease duration were associated with an increased risk of OSAHS in our population. While some studies have reported a relationship between older age and OSAHS in patients with acromegaly, findings remain inconsistent across studies [5,10,19,22,23]. Anthropometric measurements, such as BMI, did not show statistically significant differences between groups. However, BMI is a well-established independent risk factor for OSAHS [24], and its role is evident in both patients with acromegaly and those without the condition [19].

Cervical circumference, however, was significantly higher in patients with OSAHS and correlated with OSAHS severity. Patients with acromegaly generally have larger cervical circumferences compared to individuals without acromegaly, likely due to hormonal stimulation [3,25,26].

Hormonal factors are critical in the development of OSAHS [19]. In this study, patients with acromegaly and OSAHS had higher GH and IGF-1 levels than those without OSAHS. Elevated GH and IGF-1 levels promote the growth of connective, epithelial, and skeletal tissues, leading to airway narrowing [13,11,27,28]. While a definitive relationship between hormone levels and apnea severity has not been established, our study demonstrated a positive correlation between elevated hormone levels and OSAHS occurrence. This finding is consistent with the multicenter study by Vouzouneraki et al., which found that patients in the highest IGF-1 quartiles had a higher risk of OSAHS [10]. Furthermore, it supports the notion that reducing hormone levels through treatment alleviates OSAHS symptoms [11,19,22,29].

Strengths of the study

Given the high prevalence of OSAHS in patients with acromegaly, this multicenter study provides significant insights into the presentation of this comorbidity and its association with specific patient characteristics. Additionally, the identification of hormonal levels (GH and IGF-1) and related anatomical changes enhances our understanding of OSAHS severity, offering valuable evidence on the pathophysiological mechanisms that should be considered when evaluating patients with acromegaly. Consequently, polysomnography (PSG) is recommended for the diagnosis and appropriate management of OSAHS when present.

Study limitations

This study has several limitations that should be considered when interpreting the results. First, its retrospective design introduces the possibility of selection bias and is subject to the inherent limitations of the quality. Second, while significant associations between hormonal factors and the presence of OSAHS were identified, the observational nature of the study precludes establishing causal relationships. Furthermore, the lack of longitudinal data prevented an evaluation of how acromegaly treatment might influence the progression of OSAHS. Finally, the study population was limited to patients included in the RAPACO registry, which may restrict the generalizability of the findings to other populations with different demographic and clinical characteristics.

## Conclusions

OSAHS is a prevalent comorbidity in patients with acromegaly, with hormonal factors such as elevated IGF-1 and GH levels playing a critical role in its pathogenesis. This study reaffirms that higher hormone levels are associated with greater OSAHS severity, highlighting the need for comprehensive management of both conditions. Routine polysomnography (PSG) is recommended following an acromegaly diagnosis to ensure timely identification of OSAHS, which often presents with overlapping clinical features.

Early diagnosis and intervention are essential to improve respiratory outcomes and mitigate associated cardiovascular risks. If OSAHS is confirmed, targeted treatment should be initiated, and follow-up PSG should be conducted throughout the course of acromegaly management. These steps are crucial for optimizing patient outcomes and addressing the significant burden of this comorbidity in acromegaly. Future studies should investigate whether effective biochemical control directly reduces OSAHS prevalence and severity, further refining the approach to integrated care.

## References

[1] Lugo G, Pena L, Cordido F (2012). Clinical manifestations and diagnosis of acromegaly. Int J Endocrinol.

[2] Pazarlı AC, Köseoğlu Hİ, Kutlutürk F, Gökçe E (2019). Association of Acromegaly and Central Sleep Apnea Syndrome. Turk Thorac J.

[3] Colao A, Ferone D, Marzullo P, Lombardi G (2004). Systemic complications of acromegaly: epidemiology, pathogenesis, and management. Endocr Rev.

[4] de Pablos-Velasco P, Venegas EM, Álvarez Escolá C (2020). Diagnosis, treatment and follow-up of patients with acromegaly in a clinical practice setting in Spain: the ACROPRAXIS program Delphi survey. Pituitary.

[5] Dostalova S, Sonka K, Smahel Z, Weiss V, Marek J, Horinek D (2001). Craniofacial abnormalities and their relevance for sleep apnoea syndrome aetiopathogenesis in acromegaly. Eur J Endocrinol.

[6] Kapur VK, Auckley DH, Chowdhuri S, Kuhlmann DC, Mehra R, Ramar K, Harrod CG (2017). Clinical practice guideline for diagnostic testing for adult obstructive sleep apnea: an american academy of sleep medicine clinical practice guideline. J Clin Sleep Med.

[7] Uyar M, Davutoglu V (2016). An update on cardiovascular effects of obstructive sleep apnoea syndrome. Postgrad Med J.

[8] Mediano O, González Mangado N, Montserrat JM (2022). International consensus document on obstructive sleep apnea. Arch Bronconeumol.

[9] Pivonello R, Auriemma RS, Grasso LF (2017). Complications of acromegaly: cardiovascular, respiratory and metabolic comorbidities. Pituitary.

[10] Vouzouneraki K, Franklin KA, Forsgren M (2018). Temporal relationship of sleep apnea and acromegaly: a nationwide study. Endocrine.

[11] Parolin M, Dassie F, Alessio L (2020). Obstructive sleep apnea in acromegaly and the effect of treatment: a systematic review and meta-analysis. J Clin Endocrinol Metab.

[12] Acevedo K, Aguilar-Pacheco PE, Arellano-Montaño S (2010). Primer reporte del registro nacional de acromegalia: programa «Epiacro» (Article in Spanish). Rev Endocrinol Nutr.

[13] Castellanos-Bueno R, Abreu-Lomba A, Buitrago-Gómez N (2021). Clinical and epidemiological characteristics, morbidity and treatment based on the registry of acromegalic patients in Colombia: RAPACO. Growth Horm IGF Res.

[14] Ceballos-Delgado Y, Carvajal R, Buitrago Gómez N (2021). Efectividad de la terapia con análogos de somatostatina sobre el control de pacientes con acromegalia en un centro de alta complejidad, Cali-Colombia (Article in Spanish). Rev Colomb Endocrinol Diabetes Metab.

[15] Chemla D, Attal P, Maione L (2014). Impact of successful treatment of acromegaly on overnight heart rate variability and sleep apnea. J Clin Endocrinol Metab.

[16] Cao W, Wang X, Luo J, Huang R, Xiao Y (2021). Impact of obstructive sleep apnea on cardiovascular risk in patients with acromegaly. Sleep Med.

[17] Hernández-Gordillo D, Ortega-Gómez Mdel R, Galicia-Polo L, Castorena-Maldonado A, Vergara-López A, Guillén-González MÁ, Torre-Bouscoulet L (2012). Sleep apnea in patients with acromegaly. Frequency, characterization and positive pressure titration. Open Respir Med J.

[18] Turan O, Akinci B, Ikiz AO (2018). Airway and sleep disorders in patients with acromegaly. Clin Respir J.

[19] Wolters TL, Roerink SH, Drenthen LC (2020). The course of obstructive sleep apnea syndrome in patients with acromegaly during treatment. J Clin Endocrinol Metab.

[20] Cortés HT, García WR, Giraldo CMG (2022). Consenso sobre definición de criterios diagnósticos, terapéuticos y de seguimiento de la acromegalia en pacientes colombianos (Article in Spanish). Rev Colomb Endocrinol Diabetes Metab.

[21] Sankri-Tarbichi AG (2012). Obstructive sleep apnea-hypopnea syndrome: etiology and diagnosis. Avicenna J Med.

[22] Herrmann BL, Wessendorf TE, Ajaj W, Kahlke S, Teschler H, Mann K (2004). Effects of octreotide on sleep apnoea and tongue volume (magnetic resonance imaging) in patients with acromegaly. Eur J Endocrinol.

[23] Davi' MV, Dalle Carbonare L, Giustina A, Ferrari M, Frigo A, Lo Cascio V, Francia G (2008). Sleep apnoea syndrome is highly prevalent in acromegaly and only partially reversible after biochemical control of the disease. Eur J Endocrinol.

[24] Vgontzas AN, Bixler EO, Chrousos GP (2003). Metabolic disturbances in obesity versus sleep apnoea: the importance of visceral obesity and insulin resistance. J Intern Med.

[25] Gadelha MR, Kasuki L, Lim DS, Fleseriu M (2019). Systemic complications of acromegaly and the impact of the current treatment landscape: an update. Endocr Rev.

[26] Amzar D, Mihaicuta S, Golu I, Balas M, Zosin I (2010). Sleeping apnea syndrome (SAS) in acromegaly and obesity. Endocr Abstr.

[27] Fatti LM, Scacchi M, Pincelli AI, Lavezzi E, Cavagnini F (2001). Prevalence and pathogenesis of sleep apnea and lung disease in acromegaly. Pituitary.

[28] Grunstein RR, Ho KY, Sullivan CE (1991). Sleep apnea in acromegaly. Ann Intern Med.

[29] van Haute FR, Taboada GF, Corrêa LL (2008). Prevalence of sleep apnea and metabolic abnormalities in patients with acromegaly and analysis of cephalometric parameters by magnetic resonance imaging. Eur J Endocrinol.

